# TCRMatch: Predicting T-Cell Receptor Specificity Based on Sequence Similarity to Previously Characterized Receptors

**DOI:** 10.3389/fimmu.2021.640725

**Published:** 2021-03-11

**Authors:** William D. Chronister, Austin Crinklaw, Swapnil Mahajan, Randi Vita, Zeynep Koşaloğlu-Yalçın, Zhen Yan, Jason A. Greenbaum, Leon E. Jessen, Morten Nielsen, Scott Christley, Lindsay G. Cowell, Alessandro Sette, Bjoern Peters

**Affiliations:** ^1^La Jolla Institute for Allergy and Immunology, La Jolla, CA, United States; ^2^Department of Health Technology, Section for Bioinformatics, Technical University of Denmark, Lyngby, Denmark; ^3^Instituto de Investigaciones Biotecnológicas, Universidad Nacional de San Martín, San Martín, Argentina; ^4^Department of Population and Data Sciences, UT Southwestern Medical Center, Dallas, TX, United States; ^5^Department of Medicine, University of California, San Diego, San Diego, CA, United States

**Keywords:** T cell, epitope, sequence similarity, immune repertoire analysis, IEDB, epitope prediction tool

## Abstract

The adaptive immune system in vertebrates has evolved to recognize non-self antigens, such as proteins expressed by infectious agents and mutated cancer cells. T cells play an important role in antigen recognition by expressing a diverse repertoire of antigen-specific receptors, which bind epitopes to mount targeted immune responses. Recent advances in high-throughput sequencing have enabled the routine generation of T-cell receptor (TCR) repertoire data. Identifying the specific epitopes targeted by different TCRs in these data would be valuable. To accomplish that, we took advantage of the ever-increasing number of TCRs with known epitope specificity curated in the Immune Epitope Database (IEDB) since 2004. We compared seven metrics of sequence similarity to determine their power to predict if two TCRs have the same epitope specificity. We found that a comprehensive *k*-mer matching approach produced the best results, which we have implemented into TCRMatch, an openly accessible tool (http://tools.iedb.org/tcrmatch/) that takes TCR β-chain CDR3 sequences as an input, identifies TCRs with a match in the IEDB, and reports the specificity of each match. We anticipate that this tool will provide new insights into T cell responses captured in receptor repertoire and single cell sequencing experiments and will facilitate the development of new strategies for monitoring and treatment of infectious, allergic, and autoimmune diseases, as well as cancer.

## Introduction

T cells are lymphocytes that play a critical role in the function of the adaptive immune system (1). Each T cell expresses a characteristic T-cell receptor (TCR) typically consisting of an α and β chain, which are formed during T cell maturation as a result of stochastic V(D)J gene recombination (2). Different TCRs are capable of recognizing different epitopes presented by major histocompatibility (MHC) class I or class II proteins on the cell surface (3). The specificity of a given TCR is dependent upon the amino acid sequence of each chain, particularly the three highly polymorphic complementarity-determining regions (CDR1, CDR2, CDR3). Broadly speaking, CDR3 directly interacts with the presented peptide, while CDR1 and CDR2 primarily interact with the MHC molecule (4). The varying antigen specificity of different TCRs allows T cells to initiate immune responses against a broad and ever-changing range of non-self entities, including infectious agents and mutated cancer cells.

TCR repertoire sequencing has emerged as an accessible and efficient approach to capture the diversity of TCRs in blood or tissue samples of an individual (5). Using different next-generation sequencing and bioinformatics approaches, TCRs can be sequenced to different levels of resolution, ranging from paired sequencing of full α and β chains to partial sequencing of the TCR β chain by itself. Single cell sequencing with targeted TCR identification, as provided by, for example, the 10x Genomics technology platform (6), identifies the gene expression state of individual T cells, along with the receptor sequences that—in principle—indicate the specificity of these T cells. All of the main TCR repertoire sequencing approaches in use today generate information about the CDR3 region in the TCR β chain, as that part of the TCR is thought to convey the most information about TCR specificity and can serve as a “barcode” to track T cells with different specificities.

While TCR repertoire sequencing can track perturbations in the composition of antigen-specific T cell populations, it does not yield information on which TCRs are recognizing which epitopes. Such determination typically requires additional targeted experiments that can be challenging to perform. The desire to determine the specificity of T cells based on their receptor sequence has led to the development of different approaches. GLIPH and GLIPH2 (7, 8) were developed to cluster large sets of TCR sequences into groups with shared specificity, but these tools do not themselves predict likely recognized epitopes. Machine learning-based models designed to predict specificity of a receptor “*ab initio*” have also been developed (9–12). While useful in many research contexts, these methods are computationally expensive and require large numbers of known TCRs and their epitopes for training, validation, and testing in order to avoid overfitting and to learn models that generalize well. Thus, there is a need to develop new approaches to identify the specificity of TCRs in repertoire sequencing data that do not require additional experimentation, can recover the specificity for many different epitopes, and are efficient enough to process current repertoire dataset sizes in reasonable times.

In the years since high-throughput TCR sequencing became commonplace, various databases have been created to collect TCR and epitope information, such as McPAS, VDJdb, and the TBAdb subset of PIRD (13–15). Here, we set out to address the challenge of predicting epitope specificity by taking advantage of the ever-growing dataset of epitopes in the Immune Epitope Database (IEDB) that have been experimentally determined to be recognized by T cells, and for which information on the specific TCR recognizing the epitope is also available (16, 17). We tested several approaches to evaluate the sequence similarity between TCRs and examined how well they distinguished if the TCRs recognized the same epitopes or not. Using an initial test set of 24,678 TCR CDR3β sequences from the IEDB, we found that a comprehensive *k*-mer matching algorithm adopted from work by Shen et al. (18) performed best. This algorithm, which we called TCRMatch, also performed well on an independent dataset and a small dataset of paired CDR3α-CDR3β sequences. TCRMatch has now been implemented as a web server tool (http://tools.iedb.org/tcrmatch/); it is also freely available for download as a standalone command-line tool (github.com/IEDB/TCRMatch).

## Methods

### Compilation of TCR Dataset

A dataset of CDR3β sequences and corresponding epitopes was compiled by querying the IEDB on May 30, 2020 for all curated TCR entries. Starting from the IEDB homepage (iedb.org), the following filters were applied: For Epitope, “Any Epitopes”; for Assay, “Positive Assays Only” and “T Cell Assays”; for Antigen, no filters for Organism or Antigen Name; for MHC Restriction, “Any MHC Restriction”; for Host, “Any Host”; and for Disease, “Any Disease.” Any records that lacked either a CDR3β sequence or at least one peptidic epitope were filtered out. CDR3β sequences were trimmed of excess flanking residues, where necessary, using a custom pHMM-based trimming tool (manuscript in preparation). This tool was based on the ImMunoGeneTics (IMGT) (19) notation of the CDR3β region, which excludes the constant N-terminal cysteine (C) residue and C-terminal phenylalanine (F) or tryptophan (W) residue; given that the exclusion of these residues is inconsistent across published CDR3β data, this step ensured uniformity among sequences from different sources. Following trimming, the dataset consisted of 24,973 receptor groups, each defined by a unique CDR3β sequence, and 993 unique peptidic epitopes. Of these 993 epitopes, 495 were recognized by only one receptor, which were excluded from the benchmarking analysis to ensure that all TCRs in the dataset had at least one other TCR recognizing the same epitope that could be identified. The final IEDB dataset consisted of 24,678 CDR3β sequences and 498 epitopes. Of the 24,678 receptor groups, 21,851 (88.5%) recognized one epitope, while 2,827 (11.5%) recognized multiple epitopes. As a control, a shuffled dataset was established that used the same CDR3β:epitope pairs from the final dataset, but shuffled the pairings between receptor groups and epitopes. The randomized dataset was analyzed against the real dataset to serve as a benchmark for the likelihood of finding true positive matches by random chance.

### 10x Dataset

A dataset containing 15,769 CDR3β sequences and their epitope specificities was downloaded from a published application note by 10x Genomics (20). Like the IEDB dataset, all 10x sequences were run through a custom trimming tool (manuscript in preparation) to ensure adherence to IMGT guidelines. Any CDR3β that did not recognize an epitope found in the IEDB was excluded in order to ensure successful predictions were possible for all sequences. Following this filtering step, the final dataset consisted of 3,218 CDR3β sequences recognizing one or more of 18 unique epitopes.

### Similarity Metrics

Seven scoring metrics were tested to measure similarity between all possible pairs of CDR3β sequences in the IEDB dataset (Table 1). We used Parasail (21) to carry out Needleman-Wunsch pairwise alignments using the BLOSUM62 substitution matrix. An open-gap penalty of −7 and extend-gap penalty of −1 were used in tandem with default scoring settings. From these alignments, we generated the metrics Identity Alignment, Identity Long, Identity Short, and Alignment Score. “Identity” metrics were calculated by dividing the number of exact matches by either the length of the overlap between the aligned sequences (Identity Alignment), the length of the longer sequence, where applicable (Identity Long), or the length of the shorter sequence, where applicable (Identity Short). Alignment Score was calculated by dividing the score of the optimal alignment by the length of the alignment overlap. We also increased the Parasail gap penalties to −50 and −20 (open- and extend-gap, respectively) to test whether precision and recall improved when gaps were reduced for each of the four aforementioned metrics; however, we did not find any significant change in performance. Levenshtein distance, also known as edit distance, was measured between sequences using an open source Python implementation of the algorithm (https://pypi.org/project/python-Levenshtein). TCRdist was adapted from work published by Dash et al. (11). TCRMatch was implemented from work by Shen et al. (18).

**Table 1:** Description of the scoring metrics used to identify receptors with identical epitopes.

**Metric**	**Description**
Alignment Score	Alignment score divided by length of alignment
Identity Alignment	Percent identity within length of alignment
Identity Long	Percent identity within length of longer sequence
Identity Short	Percent identity within length of shorter sequence
Levenshtein	Minimum number of edits (substitutions, insertions, and deletions) necessary to transform one sequence into another
TCRdist	Similarity-weighted mismatch distance between two sequences
TCRMatch	Comprehensive comparison of all possible *k*-mers using BLOSUM62 observed frequency matrix

The TCRMatch algorithm relies on a modified version of the BLOSUM62 observed frequency matrix for amino acid substitutions (18). The two sequences being tested for similarity, seq1 and seq2, are split into sets of *k-*mers, beginning with *k* = 1 and incrementing by 1 up to the length of the sequences, or the shorter sequence if seq1 and seq2 differ in length. For each value of *k*, all possible combinations of *k-*mer pairs between the seq1 set and seq2 set are compared and assigned a similarity value derived from the values of the transformed BLOSUM62 matrix. For *k* = 1, values for each amino acid pair are looked up and summed. For *k* > 1, each amino acid of each seq1 *k-*mer is compared to the amino acid at the same position in the seq2 *k*-mer, and the matrix values are multiplied together prior to being summed across all possible *k*-mer pairs. The sums from each value of *k* are added together and divided by the normalization factor, $\sqrt{TCRMatch\left(seq1,\;seq1\right)\;\times \;TCRMatch\left(seq2,\;seq2\right)}$, to yield a score between 0 and 1, where 1 signifies a perfect match (i.e., seq1 = seq2).

### Evaluation of Prediction Performance Using Precision and Recall

To test a given similarity metric, it was applied to compare each of the 24,678 CDR3β sequences to all other CDR3β sequences in the same dataset. A range of similarity cutoffs were employed for each metric, and CDR3β sequences surpassing the similarity cutoff (“matches”) were considered true positives (TPs) or false positives (FPs) based on whether the matching CDRs recognized the same epitope or not. A TP was defined as an epitope recognized by the match sequence that was also recognized by the input CDR3β. Conversely, a false positive (FP) was defined as a match epitope that was not recognized by the input CDR3β. For a given similarity cutoff, precision was defined as TP/(TP+FP), while recall was defined as the fraction of epitopes recognized by input CDR3β sequences for which a matching CDR3β sequence with the same epitope was identified.

### Bootstrapping Approach to Compare Metrics

To compare the performance of similarity metrics, we simulated 100 datasets by sampling (with replacement) 24,678 CDR3β sequences from the IEDB dataset. Each sampled sequence was scored for similarity to the remaining 24,677 sequences using the algorithms Identity Long, TCRMatch, TCRdist, and Alignment Score. Precision and recall were calculated at each score threshold for each method. For each precision-recall curve, we calculated the area-under-the-curve (AUC) for the recall range of 0 to 0.5, yielding a distribution of 100 AUCs for each method. The AUCs were compared in pairwise fashion between TCRMatch and each of the other methods, and the fraction of the 100 comparisons in which the AUC of TCRMatch was exceeded by the AUC of the other method (i.e., the *p*-value) was determined.

### Assembly of Paired CDR3α-CDR3β Dataset

Using the same starting IEDB query as was used to compile the benchmark CDR3β dataset, we filtered out all returned TCRs that did not contain both a CDR3α and a CDR3β sequence. As above, we used a custom pHMM-based trimming tool (manuscript in preparation) on all CDR3β sequences to ensure adherence to the standard ImMunoGeneTics (IMGT) (19) notation of the CDR3β region. Similarly, for CDR3α sequences, we carried out a simple trimming step to remove a C residue from the first position and an F or W residue from the last position if both residues were present at the specified positions. To avoid the influence of exact matches, we removed any TCRs that shared an identical CDR3α or CDR3β sequence. Finally, as before, we excluded any TCRs that recognized a unique epitope in order to ensure that all TCRs would have at least one potential true positive match. After these filters were applied, the final paired CDR3α-CDR3β dataset contained 2,656 unique TCRs recognizing a total of 272 unique epitopes.

## Results

### Assembly of TCR Dataset With Known Epitopes for Cross-Validation From the IEDB

We used the IEDB (16) to retrieve T cell receptors and their known epitope specificities to serve as our benchmark dataset (see Methods). Receptors were assembled into groups defined by a unique CDR3β sequence found in the IEDB that had one or more corresponding epitopes. Our goal was to evaluate algorithms that take a query CDR3β as an input, identify similar CDR3β sequences in the database, and determine how well the similarity in CDR3β predicts that different TCRs recognize the same epitope. Given that goal, we excluded epitopes for which only one CDR3β was retrieved. All told, our IEDB dataset contained 24,678 receptor groups, each with specificity for one or more known epitopes.

### Selection of Similarity Metrics

We set out to identify established sequence similarity metrics that would be most applicable for comparing TCR sequences (Table 1). We identified four metrics based on pairwise alignment of CDR3β sequences, one on edit distance, one on similarity-weighted mismatch distance, and one on *k*-mer composition. Alignment-based methods are an established approach to measuring similarity between amino acid sequences, including TCRs (22). We carried out Needleman-Wunsch pairwise alignments for all sequence pairs and computed four similarity scores: Alignment Score, Identity Alignment, Identity Long, and Identity Short. Similarly, Levenshtein distance, which has been used previously for similarity-based clustering of TCR sequences (23), was calculated for each pair of TCR sequences. TCRdist was implemented from work by Dash et al. (11); they and others have demonstrated its utility for TCR clustering (22). Finally, the TCRMatch algorithm was implemented based on the work of Shen et al. (18) and used to calculate similarity scores for all IEDB pairs. This algorithm has been shown to be effective in identifying MAIT cell TCRs from unknown sequences (24).

### Establishment of Performance Evaluation Metrics

**Figure 1:**
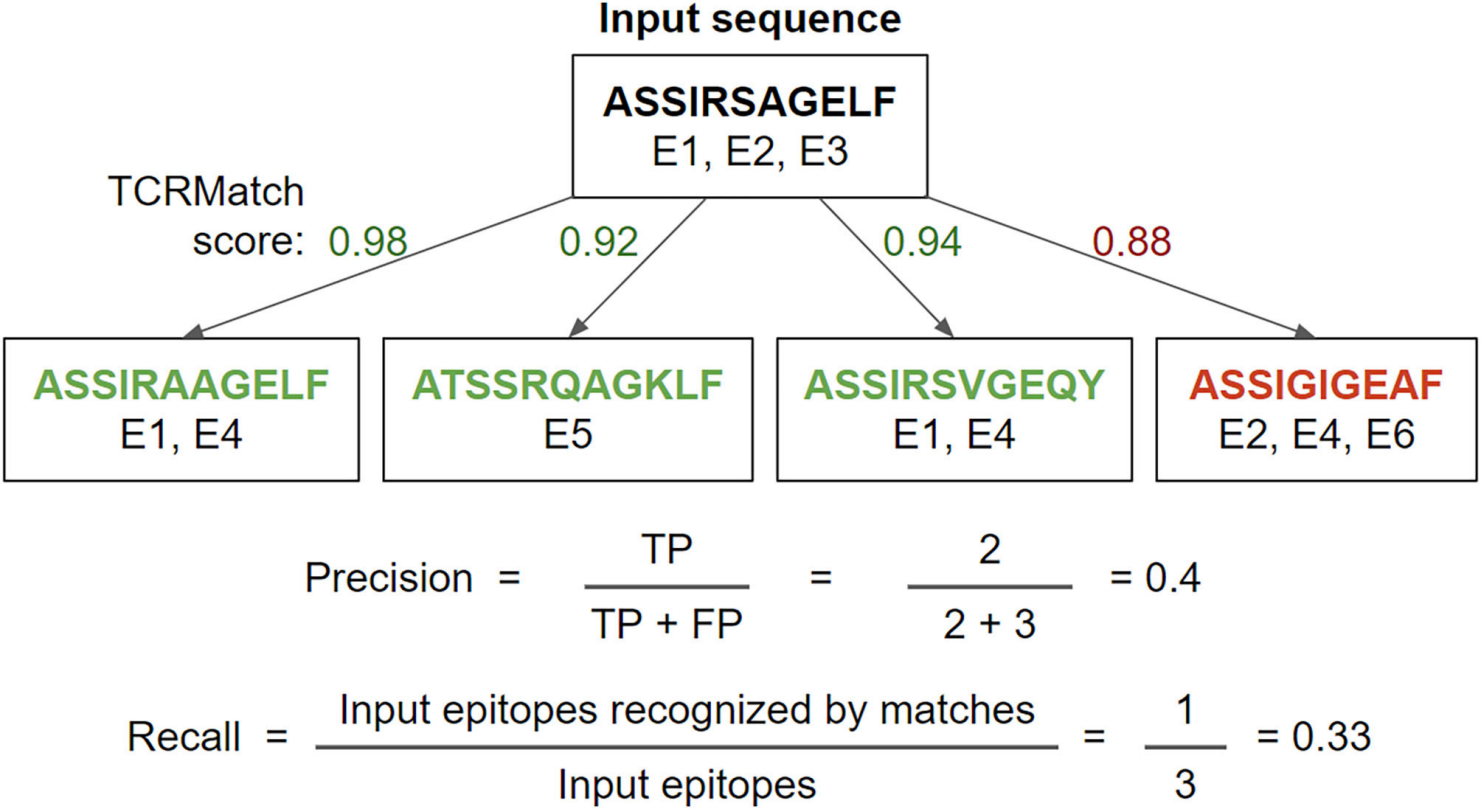
Example precision and recall calculation. Using a TCRMatch score cutoff of 0.9, the input sequence matches 3 out of 4 tested sequences. Of the 5 epitopes recognized by the 3 matches, 2 epitopes (E1, E1) are shared with A (true positives, TP) and 3 epitopes (E4, E5, E4) are not shared with A (false positives, FP), resulting in a precision of 2/5. Of the input sequence's three epitopes (E1, E2, E3), E1 is also recognized by the first and third match, while E2 and E3 are not recognized by any of the matches; therefore, recall = 1/3.

Each of the TCR sequence similarity metrics was tested for its ability to take input TCR sequences with known epitopes and match them with similar TCRs that recognize the same epitope. To measure performance, we calculated precision and recall on the epitopes recovered from such matches in cross-validation for a series of similarity cutoffs (Figure 1).

### Comparison of Algorithms on IEDB Dataset and Randomized Dataset

**Figure 2:**
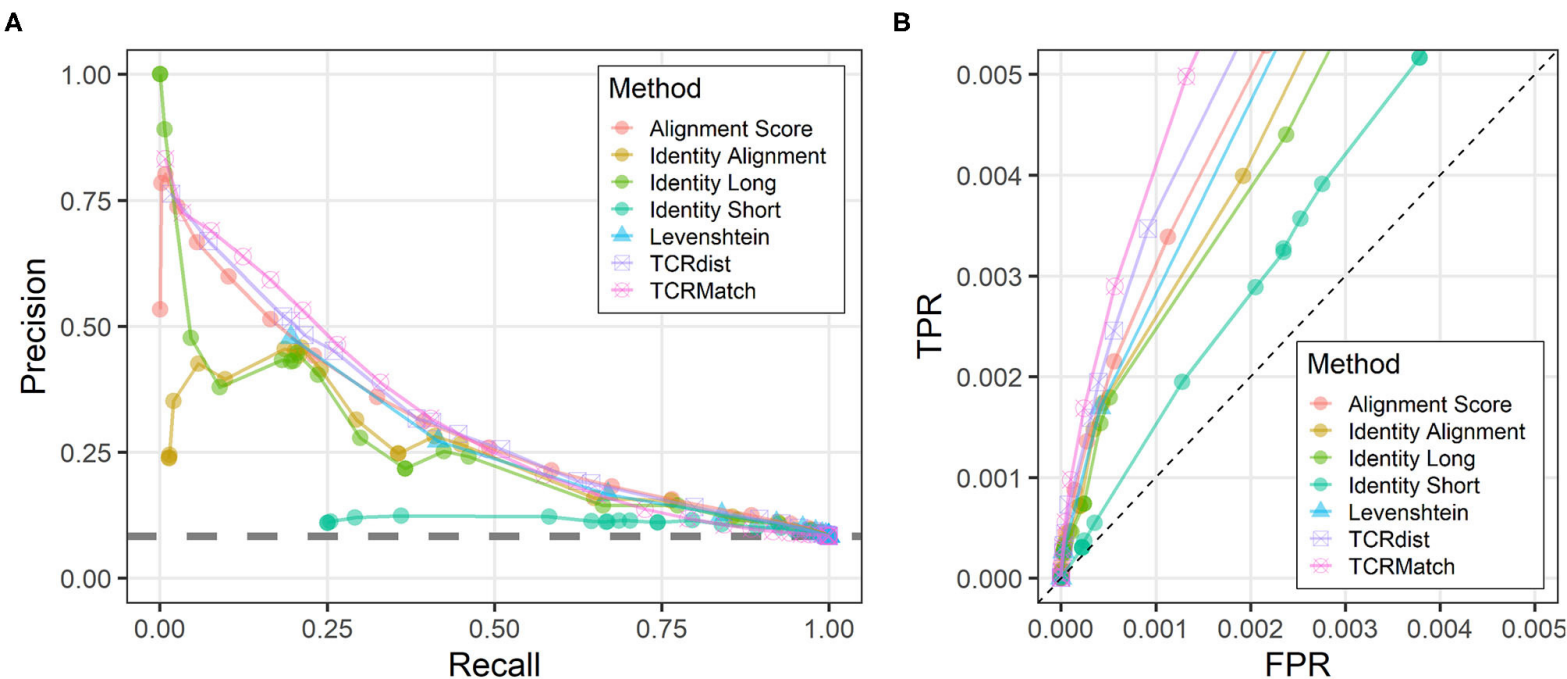
Precision-recall and ROC plots comparing different sequence similarity metrics. **(A)** Each similarity metric was evaluated at different thresholds for its ability to recall TCR sequences in the IEDB that recognized the same epitope (x-axis) and compared that to the precision at the same threshold (y-axis), which specifies the percentage of match epitopes that were also recognized by the input sequences. The gray dashed line indicates average performance on the randomized IEDB dataset, wherein CDR3β-epitope pairs were shuffled. **(B)** All similarity metrics were evaluated for their performance as measured by true positive rate (TPR, y-axis) and false positive rate (FPR, x-axis). The axis ranges of 0–0.005 show the differences in performance among similarity metrics at data points where recall < 0.5, as determined from the analysis shown in **(A)**. The dashed line indicates a random baseline for which TPR = FPR.

We tested seven similarity metrics on IEDB data and evaluated the relative success of each method using precision and recall (Figure 2A). TCRMatch was the strongest performer for recall values between 0.05 and 0.40. For example, at a similarity cutoff of 0.94, TCRMatch showed a recall of 0.213 and a precision of 0.532; in other words, for 21.3% of epitopes recognized by input TCRs, TCRMatch correctly identified a matching TCR recognizing the same epitope (recall), and 53.2% of the total match calls made at that threshold were correct (positive predictive value). At similar recall levels, ranging from 0.196 to 0.230, TCRdist, Levenshtein distance, Alignment Score, Identity Long, and Identity Alignment yielded maximum precision rates ranging between 0.441 and 0.482. The poorest performing metric was Identity Short, which at a comparable recall of 0.255 yielded a precision of 0.113; moreover, across all thresholds tested, Identity Short never exceeded 0.124 in precision. These subpar results were largely driven by perfect or near-perfect alignments of short CDR3β sequences to longer CDR3β sequences, which proved to be only weakly associated with finding a matching epitope. Starting around a recall of 0.5 and precision of 0.25, all methods except for Identity Short began to show similar precision as recall increased toward 1.

Upon inspecting the precision-recall graph, it was apparent that there were four metrics that had a precision above all others at a given recall: Identity Long (for the recall range of 0 to 0.02), TCRMatch (0.02 to 0.45), TCRdist (0.45 to 0.58), and Alignment Score (0.58 to 0.75). To compare these four metrics statistically, we performed a bootstrapping analysis (see Methods) to calculate the area-under-the-curve (AUC) for the part of the graph where precision values were at least ~3 times higher than the baseline observed in analyzing a randomized dataset (baseline precision = 0.084, dashed line in Figure 2). This corresponded to recall values between 0.0 and 0.5. This bootstrapped AUC analysis showed that the TCRMatch AUC of 0.241 (95% CI [0.235, 0.247]) was significantly higher than the AUC of Identity Long, 0.182 (95% CI [0.179, 0.190]; *p* < 0.01), TCRdist, 0.224 (95% CI [0.221, 0.230]; *p* < 0.01), and Alignment Score, 0.227 (95% CI [0.217, 0.232]; *p* < 0.01).

Analysis of the randomized dataset showed precision consistently fluctuating around 0.084 regardless of similarity metric used, a value consistent with the precision found when analyzing the non-randomized dataset without any similarity thresholds imposed. This result demonstrates that all of the tested similarity metrics are better than a randomized control at identifying receptors with the same epitope specificity, and that the best performing methods far exceed the random performance, especially at more stringent thresholds.

We further examined the similarity scoring methods for their effectiveness as measured by ROC curves (Supplementary Figure 1). By focusing on scoring thresholds found to produce recall < 0.5 (Figure 2A), we again found that all seven metrics performed better than the random baseline (Figure 2B).

### Performance Evaluation on Independent Test Dataset

Following initial tests based on the IEDB dataset, which showed TCRMatch to be the highest performing metric, we assessed the performance of all seven metrics on an independent, publicly available dataset from 10x Genomics (20). This dataset was generated using TCR repertoire sequencing of T cells found to bind to pMHC multimers presenting peptides from various viral and cancer proteins. Initially, this dataset contained 15,769 CDR3β sequences recognizing a total of 39 epitopes. The CDR3β sequences were trimmed of any flanking residues in accordance with IMGT notation (19) using a custom pHMM-based tool (manuscript in preparation) prior to analysis. To utilize this dataset for performance evaluation of a classifier trained on IEDB data, we used the subset of the 10x TCRs that recognized one of the 18 epitopes also recognized in the IEDB, resulting in a total dataset of 3,218 CDR3β sequences.

**Figure 3:**
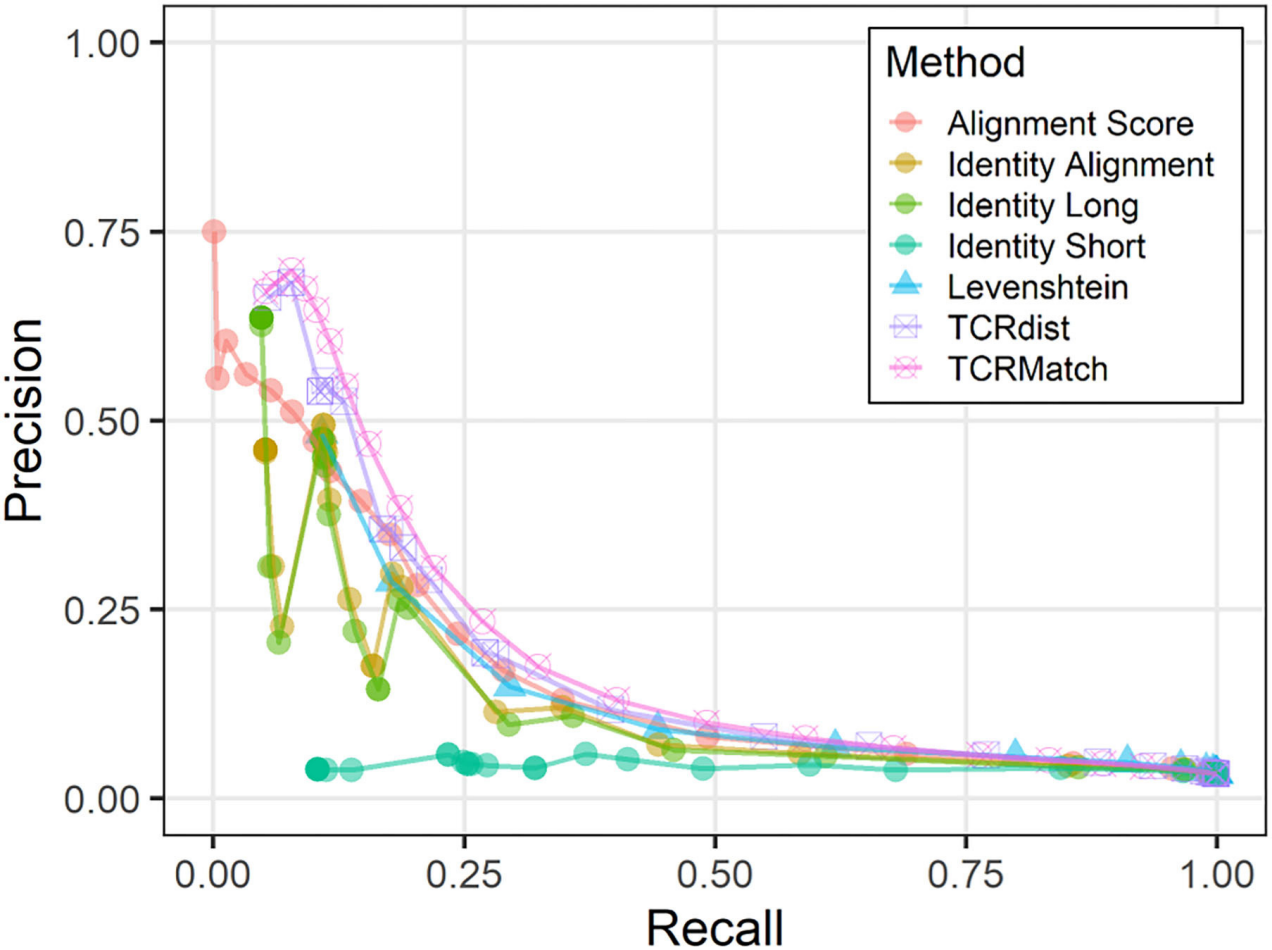
Precision-recall plots comparing performance of sequence similarity metrics on 10x dataset. Precision and recall were calculated for all seven metrics across similarity thresholds in the analysis comparing the 10x dataset against IEDB.

We used the 10x dataset as input and asked if TCRs from this dataset could have been assigned a putative epitope by searching for similar TCRs in the IEDB dataset. We observed similar results to those of the original dataset; TCRMatch leads the other methods in precision when recall is below 0.50, but the methods begin to converge as recall exceeds 0.50 (Figure 3). The precision and recall of both metrics benefited from 232 10x sequences that had exact matches in the IEDB; of these matches, 171 (73.7%) recognized the same epitope. Overall, precision was lower than when the IEDB dataset was tested against itself (Figure 2A), as expected.

### Analysis of Paired CDR3α and CDR3β Sequences

**Figure 4:**
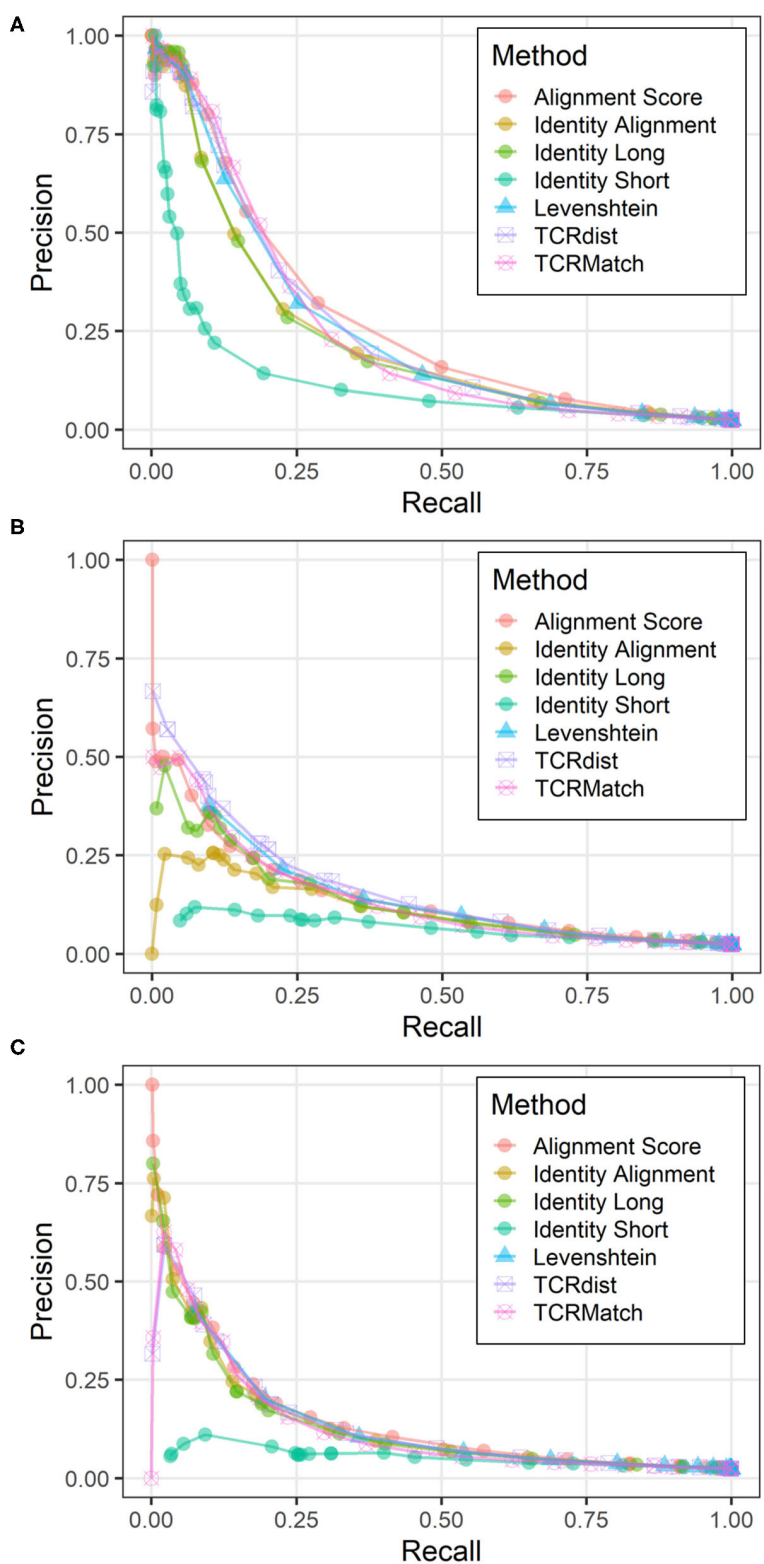
Precision-recall plots comparing performance of sequence similarity metrics on paired CDR3α-CDR3β data. Precision and recall were calculated for all seven metrics across similarity thresholds on three related datasets: **(A)** CDR3α-CDR3β pairs, **(B)** CDR3α sequences only, and **(C)** CDR3β sequences only.

To evaluate the utility of incorporating additional types of TCR data in receptor specificity analysis, we assembled a dataset from the IEDB that contained 2,656 unique CDR3α-CDR3β pairs along with their epitope specificities. Each pair was tested against the remaining 2,655 pairs using the seven similarity metrics. CDR3α sequences were compared to CDR3α sequences, CDR3β sequences were compared to CDR3β sequences, and the two scores were averaged. Using the same score thresholds as in previous analyses, we calculated the precision and recall across various levels of stringency (Figure 4A). Alignment Score produced the greatest AUC, followed by TCRdist, TCRMatch, and Levenshtein distance (Table 2). Meanwhile, Identity Short remained the worst performing metric. We also calculated precision and recall for the CDR3α and CDR3β sequences when analyzed separately (Figures 4B,C). TCRdist had the greatest AUC for the CDR3α sequences, while Alignment Score had the greatest AUC for CDR3β (Table 2). TCRMatch, meanwhile, yielded the second and fourth highest AUC for the CDR3α-only and CDR3β-only analyses, respectively. For all metrics, the AUC values for the separate CDR3α and CDR3β sets were lower than the AUC values calculated from the paired analysis.

**Table 2:** AUC 0.5 values by metric from analysis of paired CDR3α-CDR3β dataset.

**Method**	**AUC 0.5, paired CDR3α-CDR3β**	**AUC 0.5, CDR3α only**	**AUC 0.5, CDR3β only**
Alignment Score	0.235	0.115	0.120
TCRdist	0.223	0.135	0.111
TCRMatch	0.220	0.117	0.107
Levenshtein	0.213	0.079	0.075
Identity Alignment	0.199	0.083	0.112
Identity Long	0.193	0.103	0.106
Identity Short	0.090	0.041	0.034

### Implementation of a Web Server

**Figure 5:**
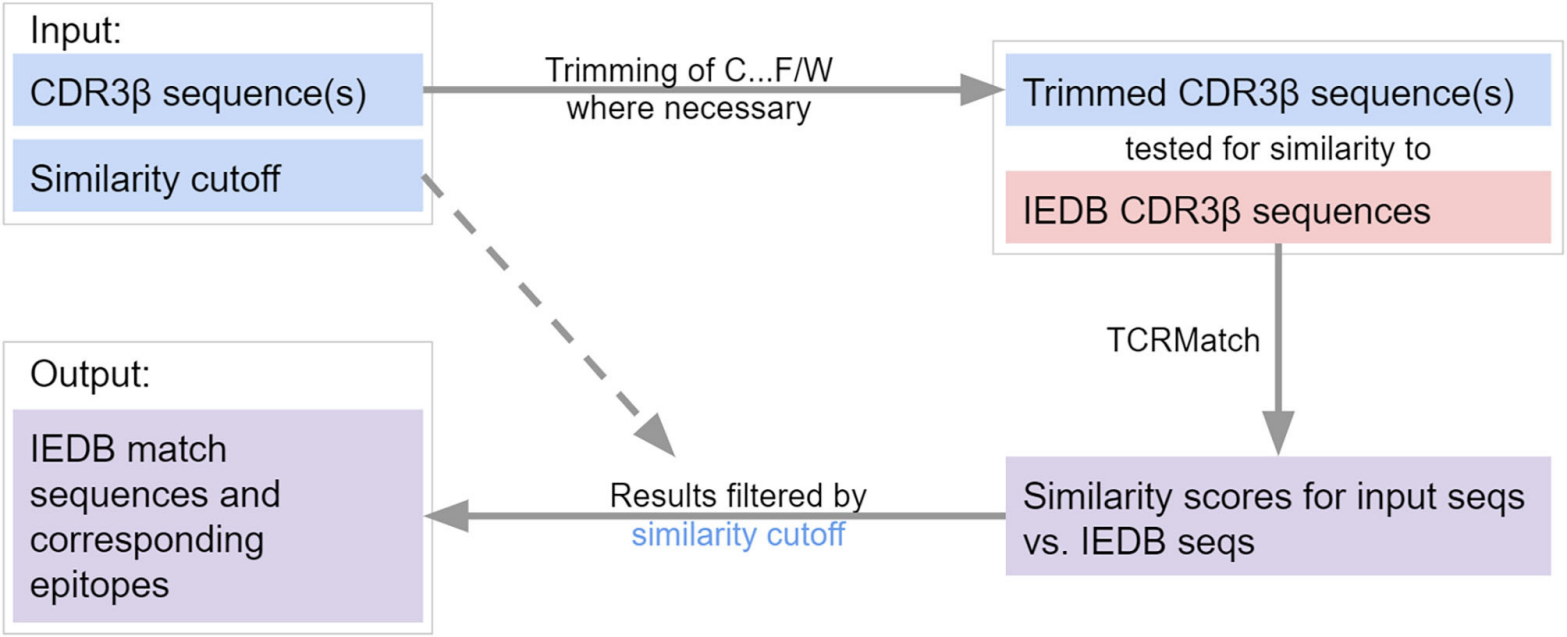
Flowchart of TCRMatch. The user provides one or more CDR3β sequences and selects a similarity cutoff. If N-terminal cysteine (C) and C-terminal phenylalanine (F) or tryptophan (W) are present, these residues are removed prior to the similarity search against the IEDB CDR3β sequences. The chosen similarity cutoff is used to filter TCRMatch's final results, which consist of matching sequences and corresponding epitopes from the IEDB.

The motivating use case for our work has been to enable users to determine what the likely epitopes recognized are for their TCR(s) of interest. We have implemented TCRMatch as a web-based tool hosted on the IEDB (Figure 5). A user can upload a set of up to 500 CDR3β sequences to query against the IEDB. Stringency can be specified by the user through the score threshold parameter. Currently, the recommended threshold for matches in TCRMatch is 0.97, which yielded a precision of 0.699 and a recall of 0.078 in the TCRMatch analysis of the 10x dataset. Lower thresholds of 0.90 and 0.84, as well as an option to return all results regardless of score, can also be chosen if more potential matches are desired. The TCRMatch algorithm is run on the back end, and results exceeding the user's score cutoff are returned in tabular format, which can be downloaded as a CSV file. For datasets exceeding 500 sequences, TCRMatch analysis is also available through VDJServer (vdjserver.org) after creating a free VDJServer account (25). Additionally, users have the option to download and run a standalone version of TCRMatch (github.com/IEDB/TCRMatch) to analyze large datasets.

## Discussion

T cells recognize epitopes presented to them by MHC molecules. Methods to identify epitopes recognized by T cells have primarily focused on the prediction of epitope binding to MHC molecules, which has been a cornerstone of immuno-informatics (26, 27). More recently, methods analyzing T-cell repertoire sequencing data have been derived to determine the specificity of a T cell based on its TCR sequence (9–12), and more tools continue to be developed (28, 29). Such methods, while powerful, are often computationally expensive and offer limited predictive power due to the data on which they were trained. While we anticipate a prominent role for such approaches when the critical mass of data becomes available, until then, approaches that can efficiently characterize the hundreds of thousands of sequences generated by TCR repertoire sequencing without requiring a trained model represent a straightforward solution to an unmet need. To meet this need, we introduce TCRMatch, a method that balances efficiency and predictive power to find relevant TCRs through sequence similarity. Our method utilizes the power of *k*-mer-based methods for computing sequence similarity with a low computational footprint. This approach allows for the processing of full repertoire sequencing datasets in an efficient and accurate manner.

The method was tested on the large amount of TCR sequence data available through the IEDB, as well as on an independent test repertoire sequencing dataset published by 10x Genomics (20). These analyses supplied evidence that the *k*-mer-based approach of TCRMatch provides a significantly higher number of relevant results compared to alignment-based or distance-based methods, in addition to being computationally efficient. Other strong performers included Alignment Score and TCRdist, the latter of which has been reported to be an effective clustering metric (11, 22). However, our analysis of paired CDR3α-CDR3β sequences demonstrated that TCRMatch is not the strongest performing metric independent of dataset. Rather, these results indicate that similarity search algorithms have strengths and weaknesses that affect their performance depending on the epitope specificities contained in the input dataset. This finding is in agreement with prior studies demonstrating the difficulty of consistently identifying epitope-specific repertoires via a single approach to grouping similar TCRs (11, 22). Further research on the underlying causes of differences in repertoire heterogeneity, such as potential differences in TCR:epitope binding modes, would provide important insights into these epitope-specific differences.

As our tool is built around the CDR3β sequences stored in the IEDB, the ability of TCRMatch to find matches is limited by the number and diversity of curated sequences available in the database. The baseline precision of 0.084 for the randomized control dataset (Figure 2A) indicates that the TCR-epitope data published to date, and collected by the IEDB, is biased toward a subpopulation of well-characterized epitopes. If the IEDB dataset containing 498 unique epitopes had an equal number of TCRs for each epitope, the expected baseline precision would have been ~1/498 (0.002). Instead, the frequency of TCRs per epitope varied considerably, with the five most common epitopes accounting for 15,243 of 28,001 (54.4%) total epitopes recognized. Indeed, the presence of heavily studied epitopes combined with the inevitability of false positives should be borne in mind when interpreting results. We have sought to minimize false positives by recommending a TCRMatch score threshold of 0.97, which yielded the peak level of precision, 0.699, with a recall of 0.078 in the 10x analysis (Figure 3). To improve this, we plan to explore ways to adjust TCRMatch results to account for an epitope's commonness or rarity, which would reduce the prominence of match TCRs recognizing a well-characterized epitope while giving a boost to those specific for rarer epitopes. If we are successful, we expect to see an increase in AUC values for the precision-recall curves (Figures 2A, 3, 4) and a stronger outperformance of the random baseline in the ROC curve (Supplementary Figure 1, Figure 2B). Further improvement will come from the IEDB curating new studies of less characterized or uncharacterized epitopes, which will expand the capability of TCRMatch to find relevant matches for input CDR3β sequences. Nonetheless, it is important to note that the imbalance of epitopes in curated TCR data has no effect on the accuracy of the tool in finding matching sequences. Furthermore, researchers interested in searching CDR3β sequences against custom databases can download and modify the TCRMatch code (github.com/IEDB/TCRMatch) to do so; this option enables users to apply the TCRMatch algorithm to the study of epitopes not yet curated in the IEDB. The standalone version of TCRMatch also enables users to generate TCRMatch scores for any two TCR sequences, independent of the IEDB, which can be applied to alternative analyses such as estimating homogeneity within a TCR repertoire.

Currently, TCRMatch identifies matches based solely on the input CDR3β sequence, which is the most closely associated with TCR specificity. However, there is much room for improvement. Our analysis of CDR3α-CDR3β pairs showed that precision-recall AUCs increase considerably when both CDR3α and CDR3β are used as inputs, as opposed to using one or the other. As more data becomes available, we anticipate that a more complex version of TCRMatch, integrating additional data including CDR3α sequences, as well as CDR1 and CDR2 sequences from both α and β chains, gene usage information (V, D, and J), and MHC restriction data, will improve the accuracy of the tool's epitope predictions.

The TCRMatch tool we develop here is well suited to complement existing tools such as GLIPH2 (8), which identifies clusters of TCRs in experimental data that likely recognize the same epitope. For example, a researcher who carries out TCR repertoire sequencing while studying a particular immune response might use GLIPH2 to discover a few hundred receptor clusters; each of those clusters can then be queried for their putative epitopes using TCRMatch, which further can enable follow-up experiments studying the epitopes driving the immune response in question. As the IEDB continues to accumulate immunological data, the quality and quantity of results produced by TCRMatch will continue to improve.

## Data Availability Statement

The original contributions presented in the study are included in the article/Supplementary Material, further inquiries can be directed to the corresponding author/s.

## Author Contributions

WC and AC: tool development, analysis, and writing the paper. SM: tool development and analysis. RV: dataset preparation and assembly. ZK-Y, LC, and AS: writing the paper. ZY: tool implementation. JG and SC: tool implementation and writing the paper. LJ: development and application of TCR data preparation tool. MN and BP: tool development and writing the paper. All authors contributed to the article and approved the submitted version.

## Conflict of Interest

The authors declare that the research was conducted in the absence of any commercial or financial relationships that could be construed as a potential conflict of interest.
