# Supplementary material for: TCRMatch: Predicting T-Cell Receptor Specificity Based on Sequence Similarity to Previously Characterized Receptors
Source: Front Immunol. 2021 Mar 11;12:640725. doi: 10.3389/fimmu.2021.640725 (PMC7991084; doi:10.3389/fimmu.2021.640725)
Supplement: Supplementary file 1 [file Image_1.pdf]

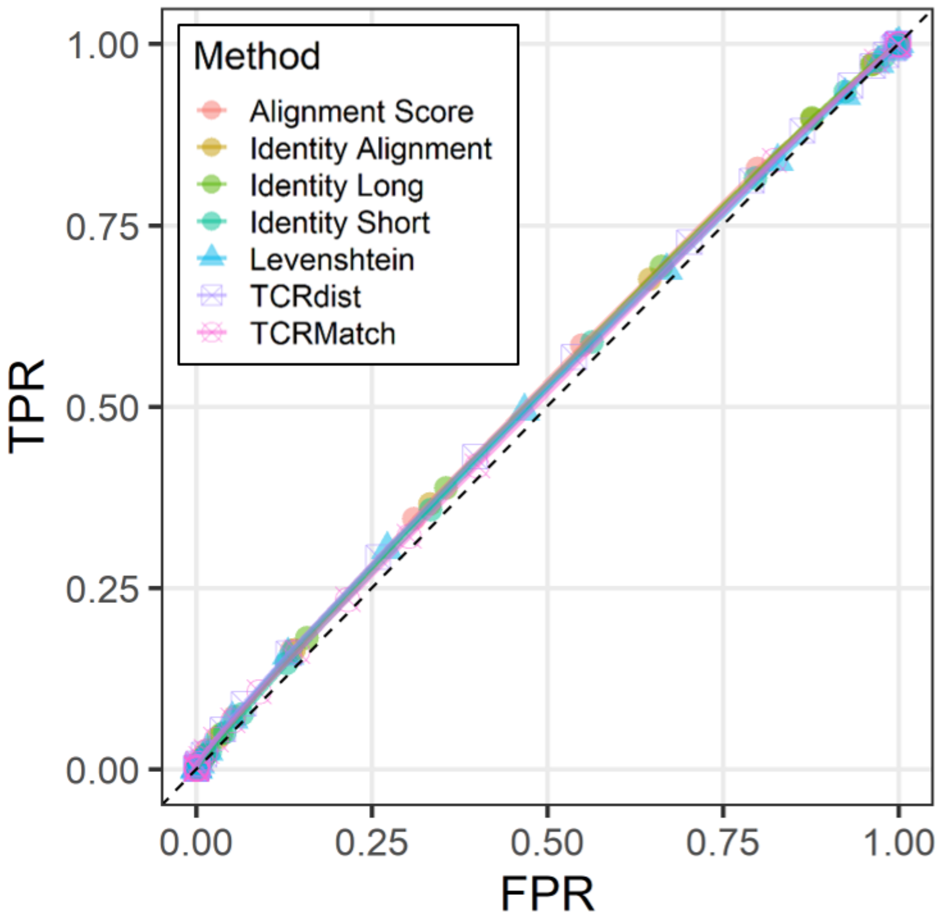

Supplemental Figure 1: ROC curves showing overall performance of similarity metrics. All similarity metrics were evaluated for their performance as measured by true positive rate (TPR, y-axis) and false positive rate (FPR, x-axis). The dashed line indicates a random baseline for which TPR=FPR.
